# The income-mortality paradox among first- and second-generation immigrants in Sweden

**DOI:** 10.1093/eurpub/ckac129.458

**Published:** 2022-10-25

**Authors:** A Miething, SP Juarez

**Affiliations:** Department of Public Health Sciences, Stockholm University, Stockholm, Sweden

## Abstract

**Background:**

This study investigates mortality differences by income among first- and second-generation immigrants and the native ancestral population in Sweden. Despite immigrants’ various vulnerabilities and the exhaustive evidence for a persistent inverse relationship between income and mortality in general Western populations, previous studies from outside Sweden demonstrated surprisingly weak income gradients in mortality among first-generation immigrants. Examining these associations among second-generation immigrants may help to understand this paradox.

**Methods:**

Swedish register data from 2002 to 2016 were used to study the association between individual income rank positions and all-cause mortality. The study population was restricted to ages 25-64 years. Based on ‘relative indices of inequality’ (RII) derived from Poisson regressions, we measured mortality differentials between the least and most deprived income rank positions stratified by nativity group and sex. Correspondingly, we assessed absolute differences in mortality between the most and least deprived by using ‘slope indices of inequality’ (SII).

**Results:**

Largest inequalities in mortality by relative income rank positions (based on RII) were found for the Swedish native ancestral population that showed on average a nearly doubled mortality risk for least compared to most deprived rank position. Immigrants disclosed weak or even nullified associations between relative income rank and all-cause mortality. Mortality inequalities by income among second-generation immigrants were substantially higher relative to first-generation immigrants but somewhat lower compared to the native ancestral population. These patterns were consistent between males and females, and confirmed by the use of SII.

**Conclusions:**

Distinct exposures to inequality structures but also first-generation immigrants’ ‘mortality advantage’ likely contribute to the considerable mortality differences by income between the studied nativity groups.

**Key messages:**

• Despite their social and economic vulnerabilities, first-generation immigrants in Sweden disclose weak associations between relative income rank and all-cause mortality.

• Second-generation immigrants in Sweden show notably higher magnitudes in income-related mortality compared with first-generation immigrants.

